# Supplementary material for: Tracking the Fidelity of Internal Neural Representations with Error-In-Variables Regression
Source: bioRxiv. 2026 Aug 3:2026.04.22.720005. Preprint. [Version 3] doi: 10.64898/2026.04.22.720005 (PMC13192658; doi:10.64898/2026.04.22.720005)
Supplement: Supplementary file 1 [file media-1.pdf]

Supplementary Figures

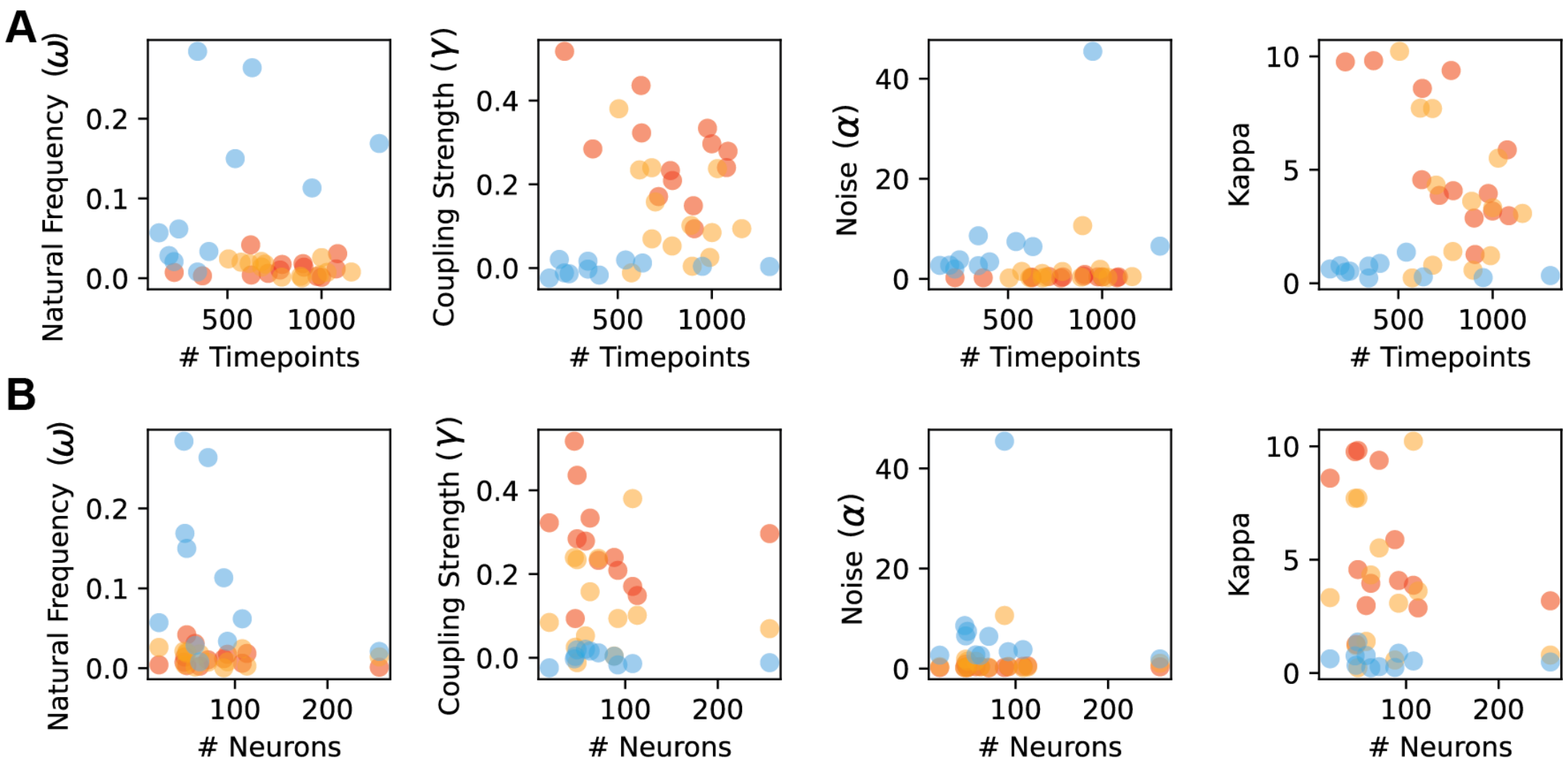

Figure S1: **Mouse ADn - correlation between model parameters and dataset dimensions** A) Oscillator model parameters and cross-validated kappa versus number of moving timepoints for each epoch of the mouse ADn experiments. Mice tended to be stationary for longer periods of the head-fixed, light condition. B) Oscillator model parameters and cross-validated kappa versus number of neurons.

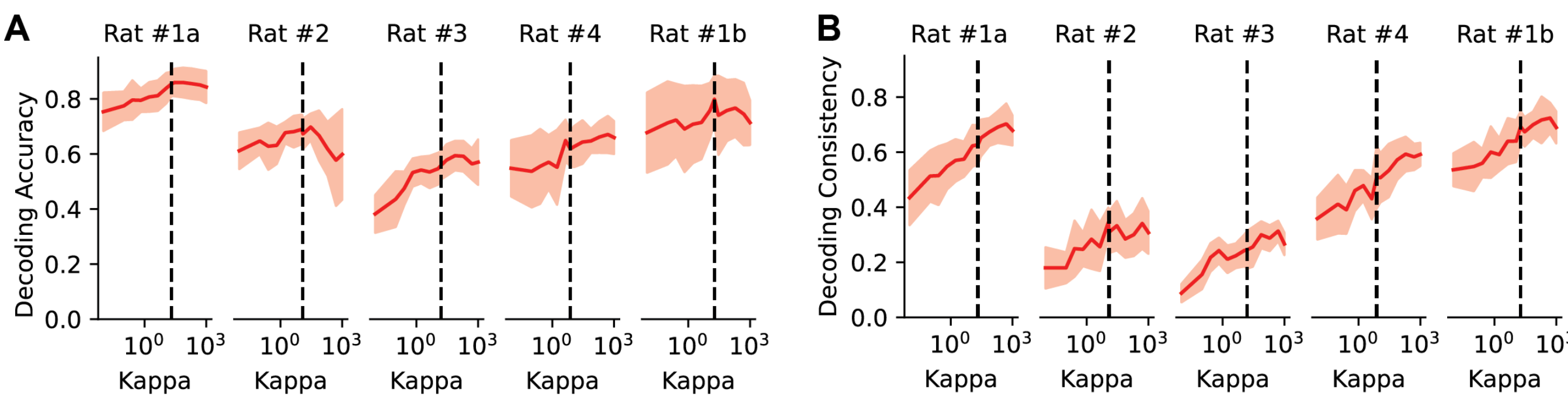

Figure S2: **Rat MEC - Decoding accuracy and consistency for light epochs** A) R^2 decoding score indicating alignment between latent estimates and observed behavior for EIV estimated tuning curves, dashed line indicates optimal κ. B) R^2 latent reconstructions from each subpopulation across range of κ, dashed line indicates optimal κ.
